# UV–Vis spectroscopy of tyrosine side-groups in studies of protein structure. Part 1: basic principles and properties of tyrosine chromophore

**DOI:** 10.1007/s12551-016-0198-6

**Published:** 2016-05-04

**Authors:** Jan M. Antosiewicz, David Shugar

**Affiliations:** 1 Division of Biophysics, Institute of Experimental Physics, Faculty of Physics, University of Warsaw, Żwirki i Wigury 93, 02-089 Warsaw, Poland; 2 Institute of Biochemistry & Biophysics, Polish Academy of Sciences, Pawinskiego 5a, 02-106 Warsaw, Poland

**Keywords:** Phenol, para-cresol, *N*-acetyl-l-tyrosine amide, UV–Vis absorption, Fluorescence, Linear and circular dichroism, Resonance Raman scattering

## Abstract

Spectroscopic properties of tyrosine residues may be employed in structural studies of proteins. Here we discuss several different types of UV–Vis spectroscopy, like normal, difference and second-derivative UV absorption spectroscopy, fluorescence spectroscopy, linear and circular dichroism spectroscopy, and Raman spectroscopy, and corresponding optical properties of the tyrosine chromophore, phenol, which are used to study protein structure.

## Introduction

Fluorescence and other spectral parameters of aromatic chromophores in proteins, tryptophan, tyrosine (Tyr) and phenylalanine, may be used as probes of protein structure. Using Tyr is an attractive choice because its chromophore, phenol, is expected to exhibit substantial responses to environmental changes (Fornander et al. 2014). We have found it interesting to review the development of structural studies of proteins based on detection of different spectroscopic features of tyrosines in the UV–Vis range, from the earliest investigations using UV absorbance measurements (Crammer and Neuberger 1943; Shugar 1952) to more recent resonance Raman (Larkin 2011; Reymer et al. 2014) or linear dichroism (Reymer et al. 2009) spectroscopy studies.

First, we recall the basic principles of different methods in UV–Vis spectrometry. We then describe properties of Tyr and related model compounds, i.e., phenol, para-cresol (*p*-cresol) and *N*-acetyl-l-tyrosine amide (*N*-Ac-Tyr-NH_2_) as revealed by different UV–Vis spectroscopies. In Part 2, we present selected applications of UV–Vis spectrometry of tyrosines to probe protein structures.

## Basic principles of UV–Vis absorption, emission, and scattering spectroscopy

### Jablonski-like diagram

**Fig. 1:**
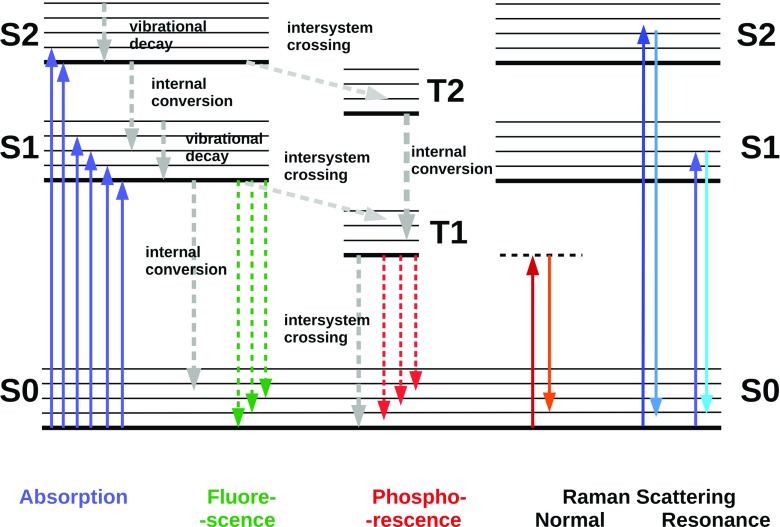
A simplified Jablonski-type energy level diagram depicting absorption, emission, and scattering of UV–Vis light, as well as nonradiative transitions. The *thick lines* represent the lowest energy levels of the electronic states, and the *thin lines* the vibrational modes. The *dashed horizontal line* represents the virtual energy level existing for a short time during light scattering

Basic principles of absorption, emission, and scattering of UV–Vis light by organic molecules can be explained using the Jablonski Diagram (Leermakers and Vesley 1964; Asher 1993; Croney et al. 2001) (Fig. 1). The horizontal lines represent singlet ground (*S*_0_) and excited (*S*_1_, *S*_2_) electronic states, triplet excited electronic states (*T*_1_, *T*_2_), and a virtual energy level (broken horizontal line) existing for a short period of time during interaction of scattered light with the molecule. Each singlet and triplet electronic state is split into vibrational and rotation energy sub-levels (for clarity, only splitting into vibrational sub-levels is presented in Fig. 1). At room temperature, most molecules are in the lowest vibrational level of the ground electronic state.

Two important types of electronic transitions for organic molecules containing systems of double bonds and nuclei of hetero-atoms are known as *π* → *π* ^∗^ and *n* → *π* ^∗^ transitions (Leermakers and Vesley 1964). They result from absorption of a photon with appropriate energy and lead from the ground to an excited singlet state. Absorption of a photon by a molecule, represented in Fig. 1 by vertical arrows directed up to the *S*_1_ or *S*_2_ level, occurs essentially instantaneously relative to nuclear motion (∼10^−15^s). The initially excited molecule may find itself in any of the allowed vibrational levels of the *S*_*n*_ electronic excited state. Subsequently, very rapidly (∼ 10^−13^ s) decays to the lowest vibrational level of the *S*_*n*_ state. If *S*_*n*_ is a higher energy state than the lowest or first excited singlet state (i.e., *n* > 1), it will quickly (∼ 10^−11^s) decay nonradiatively by a process called internal conversion to the *S*_1_ state, and finally to its lowest vibrational level. This state possesses a longer (fluorescence) lifetime, several nanoseconds to a few tens of nanoseconds, for most fluorophores of interest in biochemistry.

There are two radiative processes of return to the ground state shown in Fig. 1. Fluorescence that occurs from the *S*_1_ state and phosphorescence that occurs from the *T*_1_ state. This is known as the Kasha rule (Kasha 1950). It states that photon emission (fluorescence or phosphorescence) occurs in appreciable yield only from the lowest excited state of a given multiplicity.

The Jablonski diagram clearly shows that the wavelength of the fluorescence is independent of the wavelength absorbed, and that the emitted light is usually at longer wavelengths (lower energy) than the absorbed light (the Stokes shift). In some cases, further reduction of energy, leading to further red shift, occurs during the fluorescence lifetime by a phenomenon known as solvent relaxation. This involves re-orientation of solvent dipoles around the new excited state dipole. The reorientation favors new dipole–dipole interactions, and lowers the overall energy of the system (Inamdar et al. 1995; Improta et al. 2007).

Incident UV–Vis light may also undergo scattering by molecules present in the investigated sample. In classical scattering experiments, the incident light has a wavelength longer than the longest wavelength absorbed by the molecules. Most such incident light is scattered elastically (Rayleigh scattering), but, additionally, inelastic scattering, called Raman scattering, may be observed. Raman scattering is the phenomenon of a change in frequency of light when it is scattered inelastically from vibrational quantum states of poly-atomic molecules (Asher 1993; Robert 2009). Inelastic scattering of light of frequency *ν*_0_ may result in radiation of frequency *ν*_0_ − *ν*_*v*_ (Stokes Raman scattering) or *ν*_0_ + *ν*_*v*_ (Anti-Stokes Raman scattering), where *ν*_*v*_ is the Raman active molecular vibrational frequency. Since at room temperature high-energy vibrational modes are rather weakly populated, classical Raman spectroscopy, denoted also as non-resonance Raman scattering, is usually performed under Stokes conditions (see Fig. 1). An important advantage of Raman spectroscopy is the fact that Raman scattering of water is very weak.

In classical Raman spectroscopy, the Raman scattering is a very low probability process, and the resulting signal is very weak. However, Raman scattering can be excited at a wavelength corresponding to an absorption band of the irradiated molecule, referred to as resonance Raman scattering (Asher 1993) (RRS, also schematically presented in Fig. 1).

Different Raman spectra are observed with excitation in resonance versus non-resonance. More importantly, resonance Raman scattering can result in a signal up to 10 ^8^ times more intense than for classical Raman scattering (Asher 1993). Hence, sensitivity of resonance Raman spectroscopy can be comparable to that of fluorescence spectroscopy. Thirdly, different spectra are observed with resonance excitation within different absorption bands of a molecule. Thus, it is possible to study different segments of the molecule just by changing the excitation wavelength (Asher 1993). In addition, UV-Raman measurements of molecules in solutions excited below 260 nm are not affected by fluorescence of the chromophores (Asher 1993).

### Experimental aspects

Absorbance measurements are frequently used to determine the concentration of a chemical species in solution. One useful approach in experimental spectroscopy is absorption difference spectroscopy, where a difference absorption spectrum is calculated, i.e., the absorption spectrum of the sample minus the absorption spectrum of a reference state. This approach is useful because the difference between the sample and reference state spectra highlights only changes, simplifying data interpretation. Another useful approach is so-called derivative spectrometry (O’Haver 1982; Rojas et al. 1988; Ojeda and Rojas 2004), which uses the first or higher derivatives of absorbance with respect to wavelength for quantitative analysis. It retains all features of classical spectrophotometry: Lambert–Beer law and law of additivity (Karpinska 2004). Derivative spectra can be obtained by optical, electronic, or mathematical methods (Patel et al. 2010; Owen 1995 http://www.youngin.com/application/an-0608-0115en.pdf). An application of derivative techniques to luminescence spectrometry was demonstrated by Green and O’Hara (Green and O’Haver 1974).

With regards to emission spectroscopy, there are several important observables related to fluorescence measurements as indicators of molecular structure, dynamics, and interactions: fluorescence lifetime (defined above), fluorescence quantum yield (defined as the ratio of emitted to absorbed photons), fluorescence quenching, and rate of fluorescence decay (Lakowicz 2006). Quenching can occur by different mechanisms, e.g., deactivating collisions with solvent molecules, transfer of the excited state energy in a nonradiative way to another ‘acceptor’ molecule, and interactions with a ‘quencher’—a molecule that can deactivate the excited state either by formation of a non-fluorescent complex or by a collisional process (Croney et al. 2001).

Nonradiative energy transfer from a donor molecule to an acceptor molecule is known as FRET, the acronym of “Förster resonance energy transfer”. This transfer occurs without radiation being first emitted from one atom or molecule, and subsequently absorbed by a second atom or molecule. The resonance energy transfer is mediated by coupled Coulombic dipole–dipole interaction (Masters 2014). According to Förster (1946), the efficiency of this energy transfer, *E*, is related to the distance between donor and acceptor chromophores, *R*, and expressed as:

1 $$ \mathrm{E} = \frac{{R_{0}^{6}}}{{R_{0}^{6}}+R^{6}}.  $$

The transfer efficiency *E* is defined as the number of quanta transferred from the donor D to the acceptor A divided by the number of quanta absorbed by the donor D. *R*_0_ in Eq. 1 is referred to as the Förster radius or critical distance. For *R* = *R*_0_, the transfer efficiency *E* is half the maximal possible value. Plotting *E* as a function of *R*, it can also be noted that the transfer efficiency is most sensitive to *R* when *R* = *R*_0_. The transfer efficiency is also dependent on the relative orientation of the transition dipole moments of donor and acceptor.

FRET is a dynamic quenching mechanism. The remaining energy transfer mechanism is static quenching, which occurs when the molecules form a nonfluorescent complex in the ground state (Fraiji et al. 1992; Callis 2014).

Fluorescence and phosphorescence decay rates are influenced by solvent conditions like pH, binding of ligands, and the molecular environment of the protein of the emitting chromophore. Measurements of these rates in different conditions provides useful structural information.

Results are improved when absorption, emission, and scattering experiments are conducted with polarized light. An electromagnetic wave can be described as a two-dimensional transverse wave that has both an electric and a magnetic component, each oscillating perpendicularly to the other, and to the direction of propagation of the radiation. By convention, polarization of light means imposing some restrictions on the orientation of the oscillations of the electric component of the electromagnetic wave.

If oscillations of the electric component are confined to a given plane along the direction of propagation, then the polarization is called linear polarization, or plane polarization. On the other hand, circular polarization of an electromagnetic wave is a polarization in which the electric field of the propagating wave does not change strength, but only its direction in a rotary manner. Circular polarization is a limiting case of the more general elliptical polarization.

One type of experiment with polarized light is measurement of the linear dichroism (LD), defined as the differential absorption of light linearly polarized parallel and perpendicular to an orientation axis:

2 $$ \text{LD} = A_{\parallel}-A_{\perp}  $$

The technique of LD, besides two polarized light beams, requires some means to orient molecules in the investigated samples with respect to a distinct direction (the orientation axis). In Eq. 2, *A*_*I*_ is the absorbance for orientation *I* of the linearly polarized light. LD is a function of the wavelength of incident light.

Linear dichroism methodologies are based on the different interactions of molecules with linearly polarized light, depending on their orientation. A solution of orientated molecules displays anisotropic absorption, i.e., non-zero LD, due to an absorption vector in the molecule known as the transition dipole moment: the absorption depends (as the cosine square) on the angle between the transition dipole moment and the direction of polarization of the light. It is maximal when they are parallel, and zero when perpendicular to each other (Nordén et al. 1991).

One can introduce the reduced LD, defined as

3 $$ \text{LD}^{r} \equiv \frac{\text{LD}}{A_{\text{iso}}},  $$

where *A*_iso_ is the average absorbance of non-polarized light, usually related to *A*_∥_ and *A*_⊥_ by the relation *A*_iso_=(*A*_∥_+2⋅*A*_⊥_)/3. Assuming a single chromophore in the investigated molecules, and a single transition moment embedded within the chromophore, one can represent the LD^*r*^ as

4 $$ \text{LD}^{r} = \frac{3}{2} S \left( 3\cos^{2} \beta - 1\right),  $$

where S is a scaling factor (the orientation factor) that defines the efficiency of the macroscopic orientation. S would equal 1 for perfect orientation and 0 for random orientation, *β* is the angle that the transition moment responsible for absorption of light at a particular wavelength makes with the orientation axis (Marrington et al. 2006; Adachi et al. 2007; Fornander et al. 2014). Usually bio-molecules possess more than one chromophore, and each has more than one transition moment for absorption of light. Hence, for each wavelength a summation over chromophores (*i*) and transition moments (*u*) is required (Reymer et al. 2009)

5 $$ \text{LD}^{r} = \underset{i}{\sum}~\underset{u}{\sum}~\frac{3}{2} S \left( 3\cos^{2} \beta_{iu} - 1\right).  $$

If the LD^*r*^ is constant over the absorption band, the polarization is said to be “pure”, and no other polarizations contribute to the absorption band (Fornander et al. 2014).

Studies of polarized absorption, e.g., in oriented poly(vinyl alcohol) (PVA) films (linear dichroism), can be used to determine the absorption transition moment orientation in the investigated molecule. This orientation may also be obtained theoretically from quantum mechanical computations (Fornander et al. 2014).

Information on the transition moment orientation of a given chromophore allows one to determine the orientation of this chromophore in a larger molecular system, e.g., in a protein, from LD measurements, and knowledge of the transition moment direction in a chromophore band may help in interpreting its fluorescence spectrum in a protein in terms of orientation and dynamics (Nguyen et al. 2003).

Another application of polarized light is measurement of the circular dichroism (CD), defined as the differential absorption of left and right circularly polarized (L-CPL, R-CPL, respectively) components of plane-polarized light (Kelly and Price 2000):

6 $$ \text{CD} = A_{\mathrm{L-CPL}}-A_{\mathrm{R-CPL}}.  $$

CD occurs when a chromophore is chiral (optically active). Chirality of a chromophore may result either from its structure (intrinsic chirality), from covalent bonding to a chiral center, or by locating it in an asymmetric environment. When linearly polarized light passes through an optically active medium, it becomes elliptically polarized. Accordingly, CD, besides being expressed as the difference in absorbency of the two components [see Eq. 6], can also be expressed as the ellipticity in degrees, *𝜃*= tan^−1^(*b*/*a*), where *b* and *a* are the minor and major axes of the resultant ellipse (Kelly and Price 2000). CD is a function of the wavelength of the incident light. In most biological studies, the observed ellipticities are of the order of 10 millidegrees, i.e., the difference in absorbance between the two circularly polarized components of the incident radiation is of the order of 3 × 10^−4^ absorbance units (Kelly and Price 2000).

A further application of polarized light is detection of polarization of emitted or scattered light, both in stationary and kinetic experiments. One aspect of these effects is polarization anisotropy, defined as

7 $$ R = \frac{I_{\parallel}-I_{\perp}}{I_{\parallel}+2\cdot I_{\perp}},  $$

where *I*_∥_ and *I*_⊥_ are the fluorescence intensities of light emitted, with polarization parallel or perpendicular, respectively, to the polarization of the linearly polarized incident light (Lakowicz 2006).

## Optical properties of tyrosine chromophore

### UV absorption of tyrosine chromophore

**Fig. 2:**
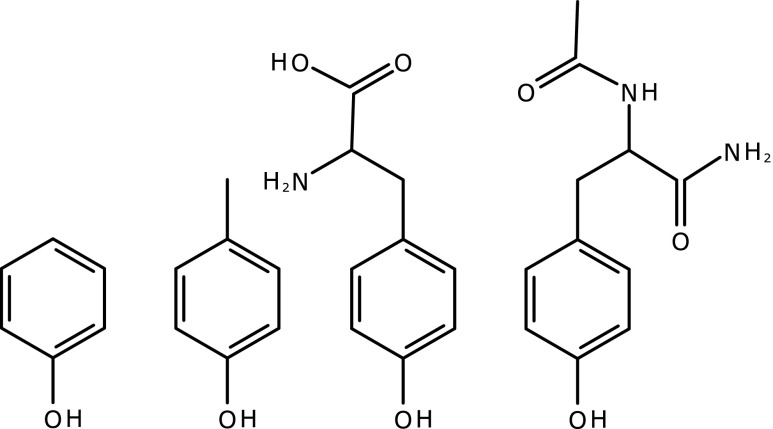
Model compounds used to study optical properties of tyrosines in proteins. From *left* to *right*: phenol, *p*-cresol, l-tyrosine, and N-Ac-Tyr-NH _2_

The Tyr chromophore, phenol, is an aromatic compound with the molecular formula C _6_ *H*_5_OH, bound to the C _*α*_ carbon in Tyr at the *para* position relative to the hydroxyl group. To model the Tyr in proteins, N-Ac-Tyr-NH _2_ (Lee and Ross 1998), or *p*-cresol (Fornander et al. 2014) may be chosen. Structural formulas for all four compounds are shown in Fig. 2.

**Fig. 3:**
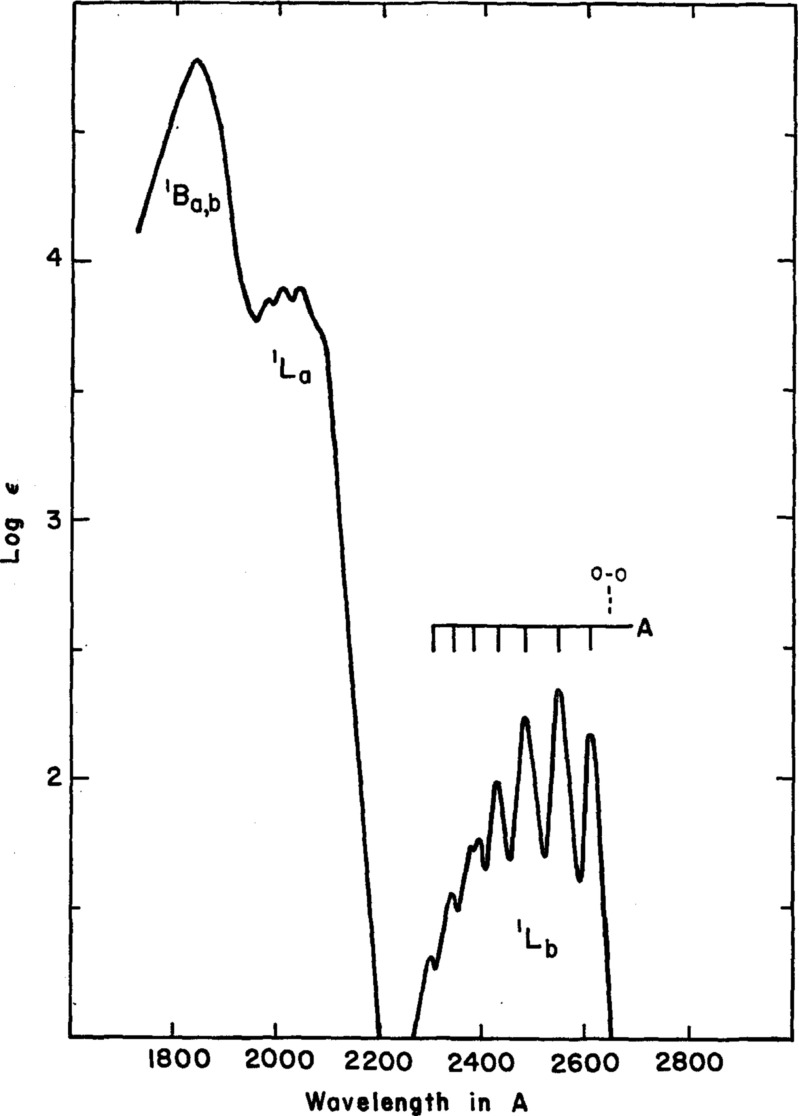
Absorption spectrum of benzene in a (non-specified) non-polar solvent. Taken from Petruska (1961)

Phenol is a derivative of benzene. Absorption (and fluorescence) spectroscopy of a benzene ring can be used to detect its presence in a larger compound and to probe its environment (Nguyen et al. 2003). Valence *π*→*π* ^∗^ excitation of benzene results in three absorption bands in the 180-270 nm region (Petruska 1961; Malkin 1992; Saik and Lipsky 1995), shown in Fig. 3 for a solution of benzene in an aliphatic-hydrocarbon solvent. They are identified with transitions from the ground electronic state (^1^ *A*_1*g*_) to the first three excited singlet electronic states that are symmetric with respect to reflection in the plane of the benzene ring. The weak 260 nm (*S*_0_ → *S*_1_) and fairly intense 205 nm (*S*_0_ → *S*_2_) bands are identified with symmetry-forbidden transitions to ^1^ *B*_2*u*_ and ^1^ *B*_1*u*_ states, respectively. The very intense 185-nm band (*S*_0_ → *S*_3_) is identified with symmetry-allowed transitions to a two-fold-degenerate, ^1^ *E*_1*u*_ state. In the labeling scheme devised by Platt (1949), these excited states are designated as ^1^ *L*_*b*_, ^1^ *L*_*a*_ and ^1^ *B*_*a*,*b*_ (or simply ^1^ *B*), respectively. The strong *S*_0_→*S*_3_ transition, being at or near the transmission limit of fused quartz, is not of much use in spectroscopic investigations (Saik and Lipsky 1995).

In the UV absorption spectrum of phenol in cyclohexane, two bands originating from *π* → *π* ^∗^ transitions are observed (Zhang et al. 2006): a primary band at 210 nm (the ^1^ *L*_*a*_ band) and a secondary band at 269 nm (the ^1^ *L*_*b*_ band) (Dearden and Forbes 1956). In ethanol, these shift to 218.5 and 271 nm (Dearden and Forbes 1956), as a result of involvement of phenol hydroxyl group in various forms of hydrogen bonding (Coggeshall and Lang 1948). Substituted phenols give similar band positions and intensities as those observed for phenol.

**Fig. 4:**
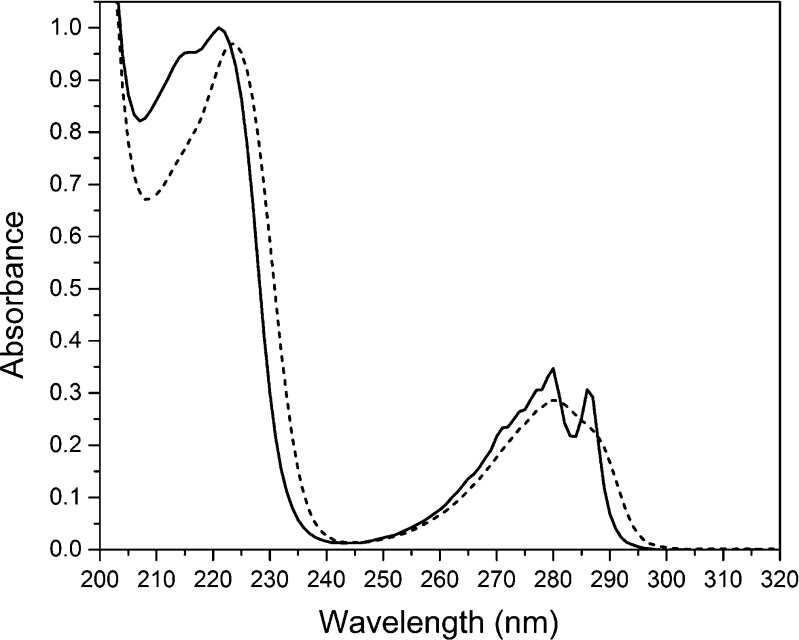
Absorbance spectra of *p*-cresol in cyclohexane (*solid line*) and methanol (*dotted line*), showing a red shift of the spectrum in methanol relative to that in cyclohexane. Taken from Fornander et al. (2014).

Figure 4 shows the change in absorption spectrum of *p*-cresol on transfer from cyclohexane (apolar, no hydrogen bonds with solvent molecules) to methanol (polar, the hydrogen bonds possible). A red shift of the L _*b*_ transition of approximately 3 nm can be clearly seen. Moreover, the highly structured vibrational peaks, distinct in the presence of cyclohexane, are not apparent with methanol as the solvent. This is due to hydrogen bonding between *p*-cresol and methanol, making the spectrum blurred as a result of the almost continuously varying composition of the environment (Fornander et al. 2014).

Absorption properties of Tyr chromophore strongly depend on pH. Ionization of the phenol group in Tyr in aqueous medium is influenced by charges of nearby amino and carboxyl groups. To avoid this effect, the Tyr residue may be incorporated into a blocked tripeptide, acetyl-Gly-Tyr-Gly-amide, where Gly-amide and *N*-acetyl-Gly serve as models of the C- and N- sides of peptide bonds along the protein chain. In such tripeptide, the phenol group has almost the largest possible solvent accessibility. Platzer et al. (2014) using ^1^H, ^13^C, and ^15^N chemical shifts, determined its pK _*a*_ as 9.76, regarded as the pK _*a*_ of a free Tyr residue.

**Fig. 5:**
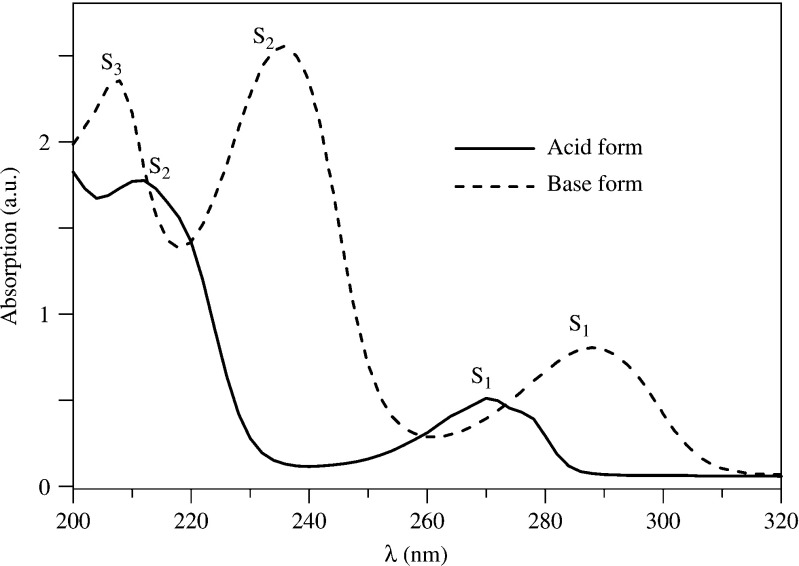
Absorption spectra of phenol (continuous) and phenolate ion (*dashed*) in water: acid-form pH = 6.0, base-form pH = 12. Taken from Pines and Rappoport (2003)

Ionization of the phenol hydroxyl in Tyr shifts the 277-nm absorption peak to 294-nm and the 223-nm peak to 240-nm (Ross et al. 2002). Figure 5 shows two lowest energy absorption bands of phenol and three lowest energy absorption bands of phenolate anion in water. The electronic transitions of the acid form are blue-shifted compared to the electronic transition of the basic form. The first two electronic transitions of phenol and phenolate ion are assigned ^1^ *L*_*b*_ (*S*_1_) and ^1^ *L*_*a*_ (*S*_2_ ) transitions (Pines and Rappoport 2003).

### Fluorescence of tyrosine chromophore

Tyr exhibits substantial fluorescence. Fluorescence is exhibited even by the most simplified precursor of Tyr chromophore, i.e., benzene. Moreover, fluorescence of benzene in aqueous medium posses clear vibrational structure (Schwarz and Wasik 1976).

Addition of substituents to the benzene ring changes its properties. The fluorescence peak of phenol in water occurs at 298 nm (Krauss et al. 1994). For comparison, Tyr emission in water occurs as a single, unstructured band with a maximum at about 303 nm (Ross et al. 2002; Lee and Ross 1998; Lakowicz 2006). Another model compound, N-Ac-Tyr-NH _2_, has a fluorescence maximum at 305 nm in water (Noronha et al. 2004).

Fluorescence properties of the Tyr chromophore may be affected by nearby molecules in several ways (Feitelson 1964; Edelhoch et al. 1969; Lee and Ross 1998). They include a hydrogen bond with a proton acceptor, a hydrogen bond with a proton donor, cation- *π* or hydrophobic interactions of the aromatic ring, and solvent polarity (Lee and Ross 1998). Tyr hydrogen-bonded to carboxyl groups or other good proton acceptors has a red-shifted emission. A good example is the shift of the fluorescence maximum of N-Ac-Tyr-NH _2_ from 303 nm in dioxane to 305 nm in water (Noronha et al. 2004).

**Fig. 6:**
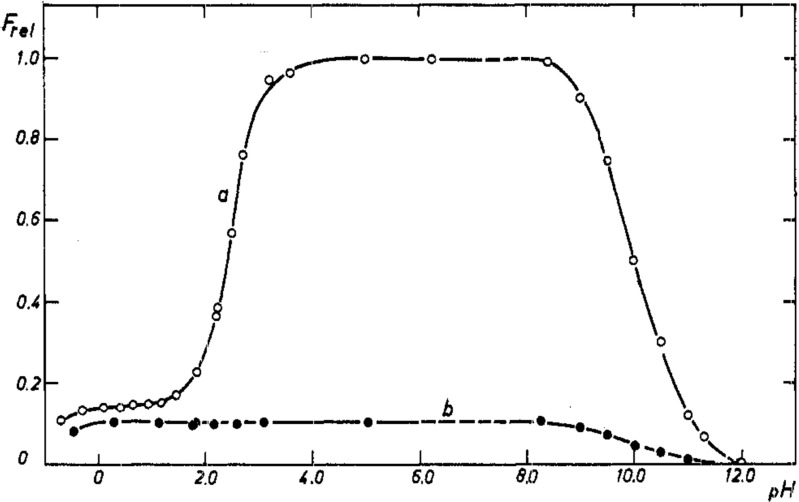
The pH-dependent fluorescence of: **a** tyrosine and **b** tyrosine methyl ester. Adapted from Feitelson (1964)

A second characteristic of the Tyr chromophore fluorescence, affected by solvent and ionization state, is its quantum yield of emission. Feitelson (1964) showed a pH-dependence of the Tyr and Tyr methyl ester fluorescence quantum yield in the pH range from 0 to 12 (see Fig. 6).

The fluorescence intensities indicated in Fig. 6 are relative values with respect to the parent Tyr at neutral pH. It can be seen that the fluorescence quantum yield of Tyr is constant in the pH range of 4–8, and decreases either on acidification or alkalization of its aqueous solution. In terms of absolute figures, this quantum yield is estimated as 0.14 (Chen 1967). Feitelson estimated it as 0.21 (Feitelson 1964). For pH > 8, the fluorescence quantum yield of Tyr decreases. Ionization of the phenolic group at pH about 10 makes this decrease very large (Edelhoch et al. 1969; Cornog and Adams 1963).

Figure 6 also shows a substantial decrease of Tyr fluorescence below pH 4 related apparently to the appearance of its non-ionized carboxyl group (pK _*a*_ of Tyr carboxyl group is about 2.3) (Feitelson 1964). It can also be seen that Tyr methyl ester has a constant, low quantum yield up to pH 8 (0.022 according to Feitelson 1964), which then decreases at pH about 12 to the value observed for free Tyr.

Quantum yield of Tyr fluorescence depends also on solvent polarity. Solvents of increasing polarity decrease the yield of tyrosyl compounds without displacing their spectra (Edelhoch et al. 1969). Addition of water to a dioxane solution quenches the fluorescence of N-Ac-Tyr-NH _2_. The quantum yield is tripled in 50 % dioxane-water and doubled in 50 % ethanol-water, relative to a neutral aqueous solution (Edelhoch et al. 1969; Noronha et al. 2004).

Quenching of Tyr chromophore can occur by a number of different mechanisms, involving the effect of acids, bases, and their salts (Feitelson 1964). It was found that, in a series of peptides with a general formula Tyr-(Tyr) _0⇔4_-Tyr, ionization of the phenolic group of a single Tyr residue in the chain is sufficient to completely quench its fluorescence (Edelhoch et al. 1969). Quenching of Tyr fluorescence by its own undissociated carboxyl group was also observed (Feitelson 1964).

A third property characterizing fluorescence of Tyr chromophore is its decay rate. Phenol and *p*-cresol, excited with *λ*_*exc*_ 284 nm, in unbuffered aqueous medium, with pH adjusted using HCl, exhibit mono-exponential fluorescence decay kinetics, observed at *λ*_*em*_ 302 nm, with invariant lifetimes *τ* (3.72–3.75 ns for phenol, and 3.36–3.37 ns for *p*-cresol) in the pH range of 2 to 7 (Laws et al. 1986). Fluorescence decay kinetics, for Tyr and N-Ac-Tyr-NH _2_, in the same pH range, is more complex. Tyr, at pH above 4.5, can be fitted with one exponential (3.73–3.76 ns), whereas lower pH values require two exponentials, with shorter *τ*_1_ of about 1.0 ns and longer *τ*_2_ decreasing from 3.53 to 2.08 ns, with decreasing pH from ∼3 to 2. Similar observations were reported more recently (Guzow et al. 2002): in the pH range of 4–8, decay of Tyr fluorescence is mono-exponential with a lifetime about 3.3 ns. In contrast, N-Ac-Tyr-NH _2_ requires two exponentials in the pH range of 3 to 6, with shorter *τ*_1_ 0.86 ns and longer *τ*_2_ about 2.20 ns (Laws et al. 1986).

The fluorescence lifetime and quantum yield of N-Ac-Tyr-NH _2_ are highly sensitive to the presence of water. For example, in pure dioxane, fluorescence decays of N-Ac-Tyr-NH _2_ are single exponentials from 23–80 ^∘^C, irrespective of excitation and emission wavelengths (Noronha et al. 2004). In dioxane-water mixtures (pH ≈6.5), at 23 ^∘^C, the decays can still be fitted with single-exponential functions up to a 70 % v/v water content, with lifetimes decreasing from 5.0 ns in dioxane to 3.2 ns in a 40:60 dioxane-water mixture. Above 70 % water, the decays can be fitted only with sums of two exponentials (Noronha et al. 2004).

### Linear dichroism of tyrosine chromophore

**Fig. 7:**
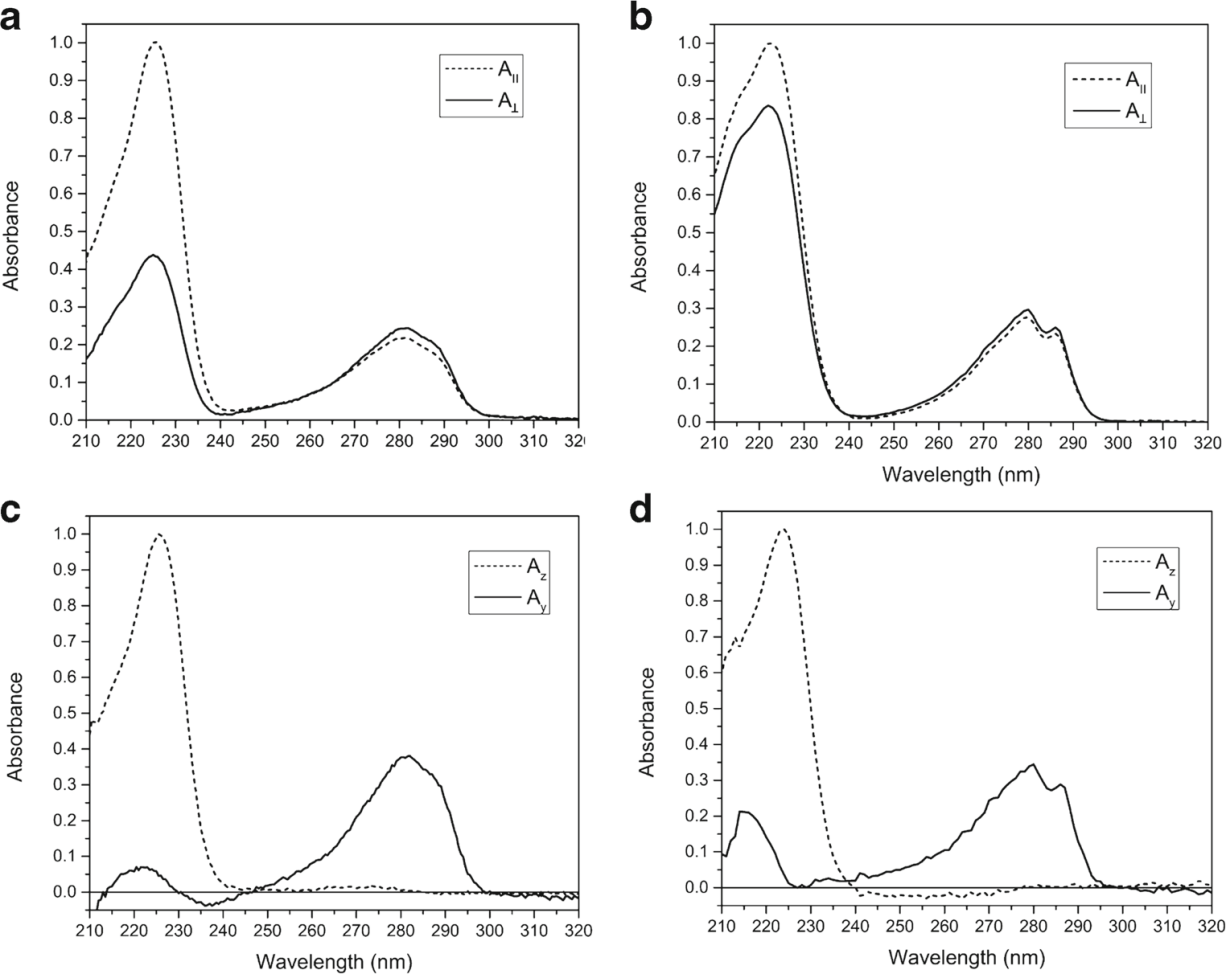
Polarized absorption spectra of *p*-cresol oriented in stretched PVA (**a**) and PE (**b**) films. The *dotted line* (A _∥_) corresponds to polarization parallel to the orientation of the stretched film, thus along the long axis (*z*-axis) of the molecule. The *solid line* (A _⊥_) shows the polarization perpendicular to the stretching orientation of the molecule (*y*- axis). Panels **c** and **d** show the calculated long- and short-axis polarizations for PVA and PE, respectively. Taken from Fornander et al. (2014)

The ^1^ *L*_*a*_ transition in *p*-cresol (absorption band ∼225 nm; see Fig. 7) lies essentially parallel with the direction defined by the methyl and hydroxyl groups (see Fig. 2). The ^1^ *L*_*b*_ transition moment, with absorption from 260 to 290 nm, is perpendicular to this direction (Fornander et al. 2014).

Panels a and b of Fig. 7 show absorption spectra of linearly polarized light by *p*-cresol oriented in stretched polyvinyl alcohol (PVA) and polyethylene (PE), respectively, normalized with respect to the parallel contribution of the ^1^ *L*_*a*_ transition, *A*_∥_. The internal difference between the parallel and perpendicular polarized absorption spectra depends on the directions of the transition moments within the molecule, and on how well the molecules are oriented in the film (Fornander et al. 2014). It can be clearly seen that the molecules have a better orientation in the PVA film compared to the PE film, because two perpendicular polarizations result in larger difference for the PVA film. Moreover, this difference is more significant for the ^1^ *L*_*a*_ transition (225 nm) than for the ^1^ *L*_*b*_ transition (280 nm), in both films.

The ^1^ *L*_*b*_ transition around 280 nm is purely polarized in both films, since no contribution from the parallel transition is seen in this spectral range (A _*z*_, dotted line in c and d in Fig. 7). The parallel transition contributes almost solely to the 225 nm absorption. However, a slight perpendicular contribution is also visible around 225 nm (A _*y*_, solid line in c and d).

Similarly to anisotropy of absorption, resulting from existence of a transition moment for absorption, there is also anisotropy of emission resulting from the existence of a transition moment for emission (Lakowicz 2006). Excitation anisotropy spectra of Tyr chromophore, in principle, could be used to establish if ^1^ *L*_*a*_ or ^1^ *L*_*b*_ is the emitting state, as done by Petrovic et al. (2013) to establish the emitting state of a single Trp chromophore in azurin. The only Tyr chromophore-related molecule for which such an investigation was done is the so-called Mannich base, 3, 5, 6-trimethyl-2(*N*, *N*’-diethylaminoethyl) phenol, for which steady-state fluorescence measurements and quantum calculations confirmed that the ^1^ *L*_*b*_ state is the lowest emitting state in the hydrogen-bonded form of this compound (Szemik-Hojniak and Koll 1993).

### Circular dichroism of tyrosine chromophore

Information concerning circular dichroism spectra of model compounds, measured in different solvents, is a prerequisite to understanding the near-ultraviolet CD bands of proteins (Strickland et al. 1972; Kelly et al. 2005). Both phenol and *p*-cresol are not chiral, and do not normally exhibit CD spectra. They can exhibit only induced CD signals (Allenmark 2003). Tyr and N-Ac-Tyr-NH _2_ possess an intrinsic chirality as the origin of their CD spectra. Their CD spectra in water have a characteristic negative peak around 275 nm, typical of many Tyr derivatives at room temperature (Horwitz et al. 1970).

**Fig. 8:**
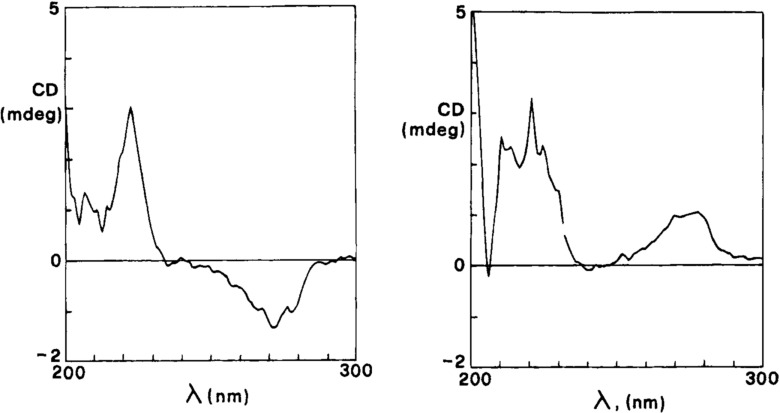
Ellipticity of phenol (left, 0.504 mM) and *p*-cresol (right, 0.552 mM) in *β*-CDx 12.5 mM aqueous solution, cell path 0.5 cm. Taken from Marconi et al. (1995)

Figure 8 shows induced circular dichroism (ICD) of phenol and *p*-cresol in *β*-Cyclodextrin (*β*-CDx) inclusion complexes (Marconi et al. 1995). The phenol spectrum exhibits a positive band with a maximum at 220 nm (*S*_0_→*S*_2_ transition) and a negative band with a maximum at 270 nm (*S*_0_→*S*_1_ transition). The spectrum of the *p*-cresol complex is quite similar. The maxima are shifted to 224 and 278 nm, respectively, in agreement with features of the absorption spectrum. A relatively strong positive band, most likely pertaining to the S _0_→ S _3_ transition, is also observed for complexes below 210 nm.

### Resonance Raman spectroscopy of tyrosine chromophore

**Fig. 9:**
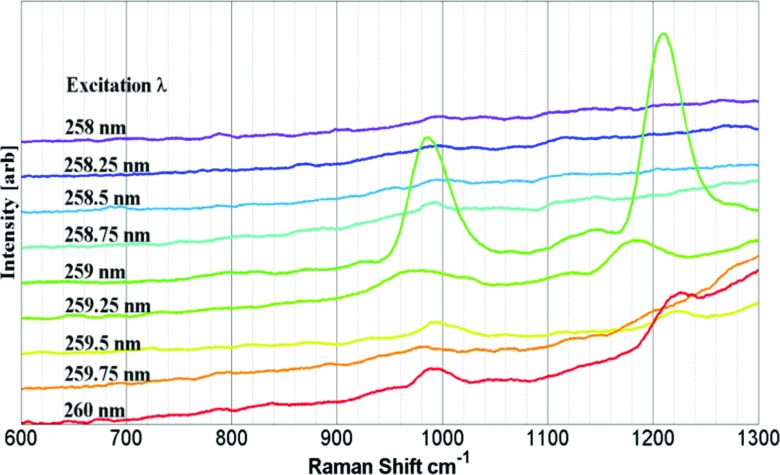
Resonance Raman spectra of liquid benzene plotted for several excitation wavelengths (each spectrum is offset). Taken from Willitsford et al. (2013)

Figure 9 shows Raman spectra of liquid benzene for several excitation wavelengths near 259 nm, separated by 0.25 nm (Willitsford et al. 2013). These excitation frequencies belong to the region of the first excited state of this molecule (^1^ *L*_*b*_). To make the comparison meaningful, each spectrum has been corrected for background, for changes in laser power as a function of tuning wavelength, and normalized to remove the inherent Raman *v* ^4^ dependence.

**Fig. 10:**
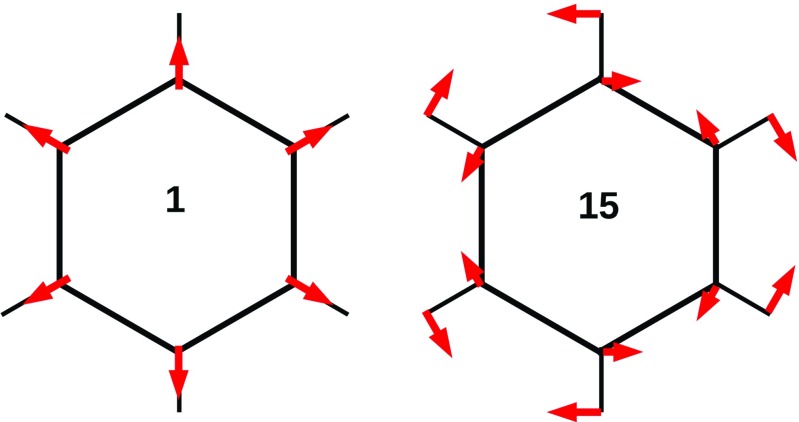
Graphic representation of the normal modes *ν*_1_ (ring breathing mode) and *ν*_15_ of benzene according to the Wilson classification

In Fig. 9 one can see resonance-enhanced Raman scatter around the 259-nm peak absorption wavelength. Two vibrational modes give intensive Raman peaks, one around 992 cm ^−1^ and the other around 1210 cm ^−1^. The frequency 992 cm ^−1^ corresponds to the totally symmetric benzene ring breathing mode (Willitsford et al. 2013; Ziegler and Hudson 1981; Asher and Johnson 1985), labeled by using the Wilson convention (Wilson 1934) with the *ν*_1_ symbol. The second resonance-enhanced Raman peak at ∼1200 cm ^−1^ was interpreted as the strengthened *v*_15_ (1150 cm ^−1^) vibrational mode according to the Wilson convention (Willitsford et al. 2013). Graphic representation of the two normal modes of benzene assigned to the enhanced Raman bands observed in the excitation with 259-nm radiation is shown in Fig. 10.

**Fig. 11:**
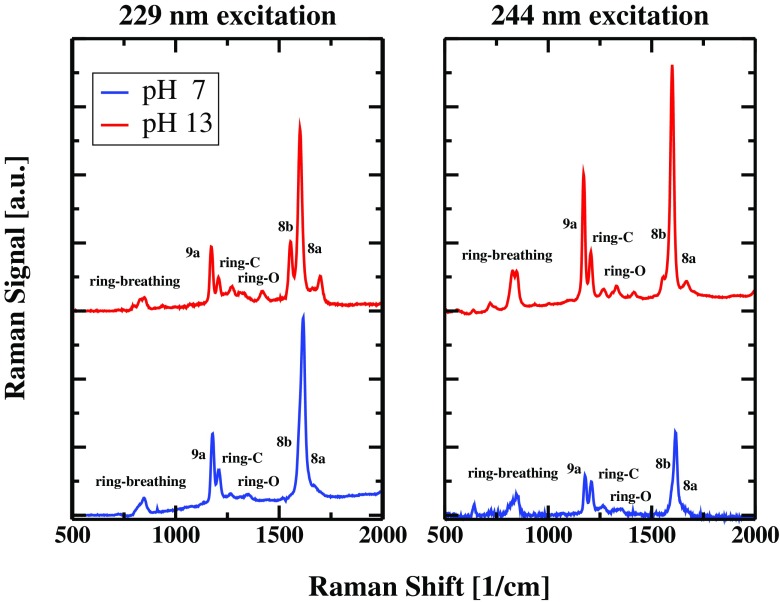
Experimental RRS spectra with 229- and 244-nm laser excitation of tyrosine in neutral and basic aqueous solutions. Figure based on experimental data, in numerical form, kindly provided by Cabalo et al. (2014)

Figure 11 presents resonance Raman scattering spectra for Tyr in water with 229 and 244 nm laser excitation, one at neutral pH and the other at pH 13, causing ionization of the Tyr hydroxyl group, obtained by Cabalo et al. (2014).

The observed Raman peaks can be assigned based on previous reports, with the most extensive being that of Rava and Spiro (1985), who presented assignment of Tyr Raman peaks at pH 7 and pH 11 with four excitation wavelengths: 200, 218, 240, and 266 nm, including relative intensities of Raman peaks for different excitation wavelengths. Some of these vibrational modes are shown in Fig. 11, where the symbols 8a, 8b, and 9a refer to the numbering scheme of benzene introduced by Wilson (1934). Moreover, a shoulder visible at 1670 cm ^−1^, in the experimental spectra, was attributed by Cabalo et al. to water molecule H-O-H bending, sharing intensity with the 8a ring C-C stretching mode.

One interesting Raman band is a doublet clearly seen around 850 cm ^−1^ in the tyrosinate spectrum with 244 nm excitation, known to be sensitive to the environment of the chromophore. The Tyr doublet at ∼850 cm ^−1^/ ∼830 cm ^−1^, which results from Fermi resonance between the Tyr ring breathing mode (*ν*_1_ Wilson 1934) and the overtone of an out-of-plane deformation (*ν*_16*a*_), is known to show large variations in relative intensities of the two components, dependent on the environment of the phenyl ring, the state of the phenolic hydroxyl group (H-bonding effects and ionization state), and the conformation of the amino acid backbone (Siamwiza et al. 1975). It can be seen in Fig. 11 that intensities of the doublet at ∼850 and ∼830 cm ^−1^ in Tyr at both excitations, and tyrosinate at 229 excitation, are substantially decreased in comparison to tyrosinate with excitation 244 nm. However, with excitation at 200 nm, this doublet is clearly revealed in the spectra of Tyr and much less visible in tyrosinate (Rava and Spiro 1985).

Another interesting Raman band is the one denoted as *ν*_8*b*_. Hildebrandt et al. (1988) reported ultraviolet resonance Raman spectra with 229-nm excitation for aqueous Tyr and some proteins, and noted that the *ν*_8*b*_ frequency decreases with the degree of Tyr H-bond donation, reaching a limiting value for deprotonated Tyr.

## Conclusions

We have discussed basic principles of absorption, emission, and scattering of UV–Vis light by molecules, and optical properties of Tyr chromophore, phenol. All of these were done in preparation for further discussion of application of UV–Vis spectroscopy of Tyr side-groups in structural studies of proteins.
